# Mesiolingual root rotation for horizontal mandibular third molar extraction: position classification and surgical simulation

**DOI:** 10.1038/s41598-017-14914-8

**Published:** 2017-10-31

**Authors:** Zhou-Xi Ye, Chi Yang

**Affiliations:** 1 Shanghai Stomatological Hospital, Fudan University, Shanghai, People’s Republic of China; 2 Department of Oral and Maxillofacial Surgery, Ninth People’s Hospital, College of Stomatology, Shanghai Jiao Tong University School of Medicine, and Shanghai Key Laboratory of Stomatology, Shanghai, People’s Republic of China

## Abstract

Extracting horizontal mandibular 3rd molars face considerable difficulty due to the large bone and adjacent tooth resistances. This study aims at evaluating the effectiveness of a novel method-mesiolingual root rotation to extract wisdom teeth of this type. In this study, 73 horizontal teeth extracted using piezosurgery were reviewed and classified based on impaction depth: position I, II, III refers to the highest portion of the crown on a level with upper 1/3, middle 1/3, lower 1/3 of the 2rd molar’s root. Based on the surgical simulations on their 3D CBCT reconstructions, traditional method(crown distal rotation) and novel method(root mesiolingual rotation) are applied. 79.17% of teeth in position I and 57.89% of teeth in position II were designed using traditional method, 83.33% teeth in position III were designed using the novel method(p < 0.05). The surgeries were performed according to the designs. Two cases in position II using traditional method were found temporary inferior alveolar nerve(IAN) injury; while only one case in position III using novel method got temporary IAN and lingual nerve injury. Our study suggested that root mesiolingual rotation is an effective method to extract the horizontal mandibular 3rd molars, especially the deep impacted ones.

## Introduction

In relation to the long axis of the adjacent tooth, the wisdom teeth could be classified based on the impacted angulation: distal-angular, vertical, mesio-angular, horizontal, as well as inverted impaction. Among them, horizontal mandibular third molars are usually deep impacted and are most difficult to extract due to the following features: ①large coronal bone resistance; ②large adjacent tooth resistance; ③the proximity to inferior alveolar canal(IAC); ④mandible fracture risk due to the deep impaction. Therefore, any unplanned surgery for horizontally impacted tooth will easily lead to excessive surgical trauma and inferior alveolar nerve (IAN) injury.

To avoid the resistance of the crown (large bone and adjacent tooth resistance), tooth lingual rotation is introduced in horizontal mandibular third molar extraction. In our previous study^1^, 87.3% of all impacted mandibular third molars were measured in lingual positions by evaluating buccal-lingual alveolar bone thickness, which indicates the valid of lingual split technique. In our another study^2^, tooth rotated in horizontal direction(root mesiolingual rotation in particular) with the application of piezosurgery^3–5^, has the potential of notably avoiding almost all adjacent tooth resistance and bone resistance.

Considering the remarkable characteristics of less trauma and fewer complications, the novel method of mesiolingual root rotation is supposed to be introduced in extraction of horizontal mandibular 3rd molars. However, though there are some studies mentioned lingual split technique^6–10^, none of them has described the details of lingual root rotation according to our knowledge. Several problems as follows are to be answered: ①Is there a comprehensive evaluation for the deep impacted mandibular 3rd molar? With the application of three-dimensional(3D) reconstruction in oral and maxillofacial surgical design^11,12^, is it possible to introduce the technique in this study to guide the surgical procedure? ②What is the key technique in the mesiolingual root rotation? ③Are there any complications after surgery using the novel method? In order to solve those problems, we designed this prospectieve study. By reviewing horizontal mandibular third molar extractions, the study aims at presenting the design procedure and novel surgical method, as well as its clinical value.

## Material and Methods

### Patients

This is a prospectieve study. This study followed the Declaration of Helsinki on medical protocol and ethics and the regional Ethical Review Board of Shanghai Ninth People’s Hospital approved the study. Each patient was informed about surgical purpose, surgical protocol, recovery period, possible complications and signed a consent form.

From January 1st 2015 to May 31th 2016, a consecutive group of 171 patients who had piezosurgery extraction of 243 complicated mandibular third molars were reviewed. After a detailed medical and dental history were gained, OPG and cone-beam computed tomography (CBCT) of the surgical site were taken in all patients. All teeth were classified and evaluated based on the Pell&Gregory classification^13,14^ and the proximity to the mandibular canals^15–17^.

The inclusion criteria is horizontal mandibular 3rd molars. The definition of horizontal impaction was based on the angulation of mandibular 3^rd^ molar axis to the mandibular 2^nd^ molar shown in orthopantomogram (OPG). In total, 56 patients with 73 teeth were included.

### Teeth position classification

**Figure 1:**
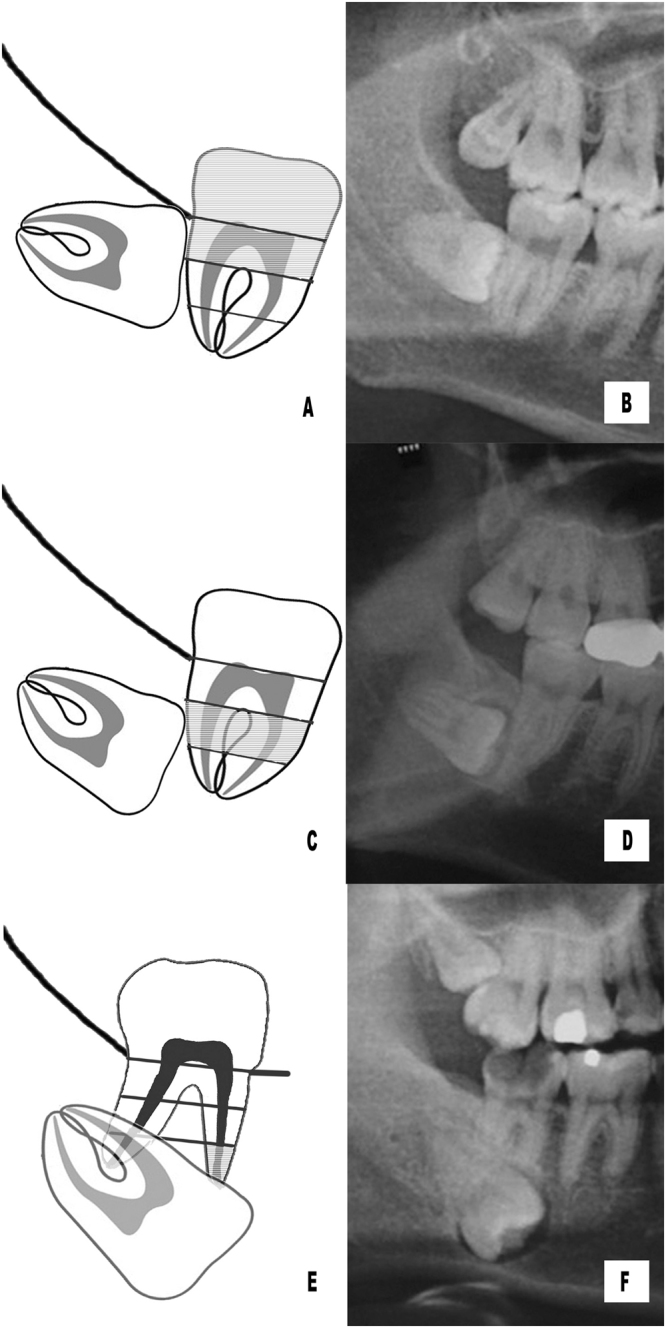
Position classification of horizontal impacted mandibular third molars. (**A**,**B**) position I; (**C**,**D**) position II; (**E**,**F**) position III.

Based on the relative impaction depth in the bone, the included mandibular third molars were classified into three types (Fig. 1):

Position I: The highest portion of the tooth crown is on a level with/above the upper 1/3 of the 2rd molar’s root (Fig. 1A,B).

Position II: The highest portion of the tooth crown is on a level with the middle 1/3 of the 2rd molar’s root (Fig. 1C,D).

Position III: The highest portion of the tooth crown is on a level with/below the lower 1/3 of the 2rd molar’s root (Fig. 1E,F).

### Surgical design and simulation

**Figure 2:**
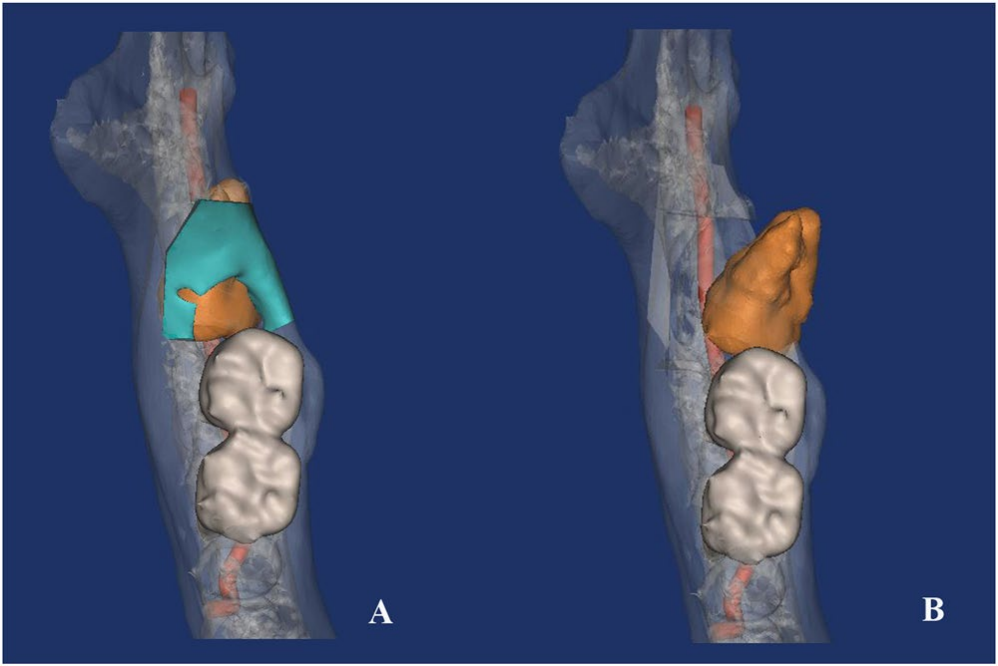
Surgical simulation using root mesiolingual rotation method. (**A**) osteotomy procedure; (**B**) root rotation direction.

The surgical design and simulation in each case includes the following steps: (1)transferring CBCT data into mimics 15.0 (Materialize Co, Leuven, Belgium) for 3D reconstruction. (2)segmenting out teeth, alveolar bone and inferior alveolar nerve. (3)simulating osteotomy and tooth rotation on the 3D model^12^ (Fig. 2).

According to the surgical simulation results, all the extraction simulations could be concluded into two types as follows: ①traditional method: remove the occlusal bone, then rotate the crown in mesio-distal direction with/without sectioning; ②novel method: remove the occlusal and lingual bone, then rotate the root in mesiolingual direction to make the whole tooth luxated.

### Surgical procedure

Under local anesthesia with 2% lidocaine, all patients were operated by one surgeon(Yang C) with 30-year clinical experience. After a full thickness flap elevated, a piezosurgical device (Silfradent, Italy) was implemented for bone removal. A curved periosteal elevator was placed on the lingual bone to improve surgical field exposure, protect the lingual nerve, as well as prevent the tooth or bone pieces slipping into the lingual soft tissue space. The extraction procedures were in accordance with the design.

**Figure 3:**
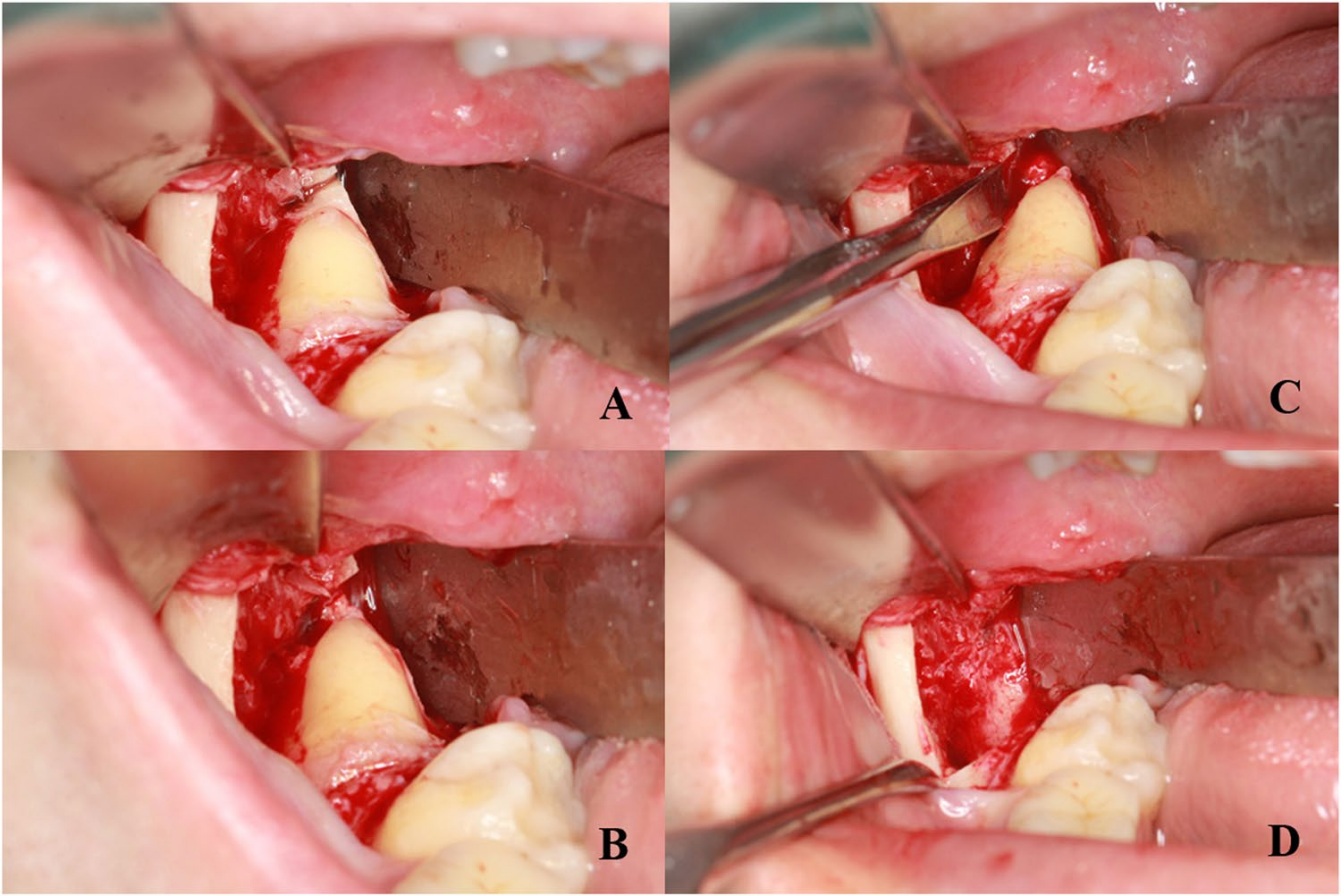
Surgical procedure using root meisolingual rotation method. (**A**) root resistance release; (**B**) root mesiolingual rotation with the elevator on the buccal side.

Piezosurgery was applied for bone removal in all the extractions. In the novel extraction, the bone resistance around the root was precisely released with piezosurgery and then the root rotate mesiolingually with the elevator located on its buccal side (Fig. 3). After extraction, the sockets were closed by 4/0 absorbable silk (Covidien, US) sutures.

### Outcome assessment

Outcome variables: success rate, operating time (from the first incision to the last suture), recovery (pain, restricted mouth opening, swelling) and the incidence of major complications. Patients were recalled 1 week, 1 month, 3 month, 6 month, 1 year after surgery.

Pain level was measured with Visual analog scale (VAS) method^18,19^. Mouth opening was measured as the inter-incisal distance at maximum mouth opening^10,19^. Swelling was measured with standard caliper from the lingual aspect of mandibular 1st molar crown to the tangent of the cheek skin^20^.

Major severe complications include 2^nd^ molar injury, lingual nerve(LN) and inferior alveolar nerve(IAN) damage, mandibular fracture, temporomandibular joint injury^21^. Sensory deficit lasting longer than 6 months is defined as permanent.

Data were collected in a spreadsheet (Excel; Microsoft Inc, Redmond, WA) and analyzed using a statistical software package SAS 8.0 (SAS institude, USA). Chi-square test and Fisher’s Exact Test are used to analyze the data, a value of p ≤ 0.05 was considered statistically significant.

## Results

In this study, there are 73 teeth in 57 patients included. Of these, 35 were female (44 teeth), and 22 were male (28 teeth). Their ages ranged from 20 to 48 years (average, 31 years). Among them, 48 teeth were in position I, with 38 of them designed to be extracted using traditional method, and 10 designed using root mesio-lingual rotation; 19 teeth were in position II, with 11 designed using traditional method, and 8 designed to rotate the root mesio-lingually; 6 teeth were in position III, with 5 designed root rotation (Table 1).

**Table 1:** Position classification of horizontal mandibular 3^rd^ molars and their extraction methods.

Classification	Extraction method		p
	Traditional method	Novel method	p < 0.05
Position I	38	10	
Position II	11	8	
Position III	1	5	

*p ≤ 0.05 is considered statistically significant.

The success rate of all extractions was 100%. The average operating-time was 15.6 min (p < 0.05). The VAS score, mouth-opening, swelling measurement in patients were 0.35 ± 0.48, 35.1 mm ± 3.7 mm, 2.39 cm ± 0.22 cm one week after surgery (p > 0.05).

Two cases in position II using traditional method and one case in position III using novel method were found temporary lower lip hypesthesia, indicating IAN injury, and recovered in 7 weeks after surgery. Among them, the case using novel method combined with temporary half tongue hypesthesia, indicating LN injury, and recovered in 12 weeks (Table 2). None of other major complications was recorded.

**Table 2:** The cases involving temporary nerve injuries.

Patient list	gender	age	Pell&Gregory classification	Proximy to the canal	method	Injured nerve	Recovery time
1	Male	52	3C	penetrate	1	IAN	7 weeks
2	Female	40	3C	contact	1	IAN	3 weeks
3	Male	42	3C	contact	2	IAN, LN	IAN injury: 4 week; LN injury: 12 weeks

IAN = inferior alveolar nerve, LN = lingual nerve.

## Discussion

Horizontal mandibular 3^rd^ molars are usually deep impacted with large bone resistance, leading to the difficulty of extractions as well as the risk of IAN injury. Besides, they occasionally locate below the cervical lines of the mandibular 2^nd^ molars, producing the risk of adjacent teeth trauma. Therefore, extracting mandibular 3^rd^ molars of this type is quite a challenge. In order to solve this problem, mesiolingual rotation is introduced^1,22,23^. However, neither the detailed application, nor the clinical effect has been discussed in literature. The purpose of this study was to introduce root mesiolingual rotation as a novel method to extract horizontal mandibular 3^rd^ molars. In this study, the novel method was implemented with piezosurgery and achieved a satisfying outcome—acceptable operating time, controlled recovery period with infrequent complications.

For mandibular 3^rd^ molars, OPG could be used to make general classifications, helping the surgical difficulty evaluation. While CBCT could show more detailed information in 3 dimensions, which had the value in surgical guidance^16,23,24^. In addition, the data of CBCT could be transferred for reconstruction and surgical simulation with the segment tools. Considering the strengths of the two radiological examinations, for complicated mandibular 3^rd^ molars (e.g. penetrate the mandibular canals, impact deeply in the bone, root curve or hypertrophy), implementation of both OPG and CBCT contributes to a better evaluation^5,17^. In this study, the 3D reconstruction of CBCT and surgical simulation based on it have been proven valid in evaluation and surgical design. The shortcomings of this computer technique are time-consuming and the requirement of thin slice interval (≤0.1 mm). The data with thick slice interval are not suitable to reconstruct smooth and precise 3D model, which will produce large deviations in surgical simulation.

Invasive extraction and less bone removal are the current notion for mandibular 3^rd^ molar extraction. Therefore, the traditional method involving the steps of crown bone removal, tooth sectioning and distal crown rotation is recommended to extract wisdom teeth. However, for horizontally impacted teeth, especially for the teeth deep impacted, the traditional extraction method leads to narrow surgical site exposure, large bone removal, in additional to higher risks of complications such as inferior alveolar nerve injury and bone fracture. In this study, we classified deep impacted teeth into 3 categories based on the impaction depth. It shows that the deeper impaction, the more frequency of choosing root mesio-lingual rotation. The reasons of choosing root rotation instead of the crown are listed as follows: ① the bone resistance around the crown is much larger than that around the root; ② the deep impacted crown always locates in closer proximity to the mandibular canal than the root; ③ the deep crown contact the adjacent tooth closely, and the root rotation could avoid the resistance.

With the application of piezosurgery, the goal of the precise and easy bone removal in alveolar surgeries is achieved. Compared with the drill device, piezosurgery has distinguished characteristics including minimally invasive, precise osteotomy, less thermal damage, as well as less soft tissue injury^4,20,25^. In the method of mesio-lingual root rotation, the key technique is the release of the bone resistance around the root. The procedure is performed by piezosurgery as follows: ①firstly remove the occlusal bone to the root level precisely^5^; ②then carefully make the osteotomy line from the root area to the bone lingual surface (Fig. 3). With the removal of the occlusal and lingual bone, the root could rotate in mesiolingual direction to make the whole tooth luxated by applying an elevator at its buccal side.

The evaluations during the extractions include the success rate and operating time. In this study, success rate was 100%, indicating the effectiveness of the two methods. The average operating time of all the extractions was adequate compared with the previous studies^18,19,26,27^. The post-operative reactions^28^ (pain, limited mouth-opening, swelling) are mild and acceptable compared with other studies of mandibular third molar extractions^10,18–20^. In the three cases of temporary IAN injury, all of the teeth located deeply and contacted or penetrated to the canals, which may be the cause of nerve injury^29^. Some studies mentioned that lingual split technique would increase the risk of lingual nerve injury^9,30,31^. In this study, a proper protection of lingual soft tissue was implemented, and only one case of temporary lingual nerve injury occurred, which demonstrated that the lingual nerve injury risk could be well controlled. The occurrence may due to the deep impaction of the tooth (below the lower 1/3 of the adjacent tooth root).

It is suggested that root mesiolingual rotation is a valid method, especially for extracting deep horizontal mandibular 3^rd^ molars. However, in extracting teeth with wide root furcation, this method has its limitation because of the large bone resistance around the roots. Therefore, tooth sectioning method is recommended in this situation. A potential limitation of this study is the possibility of referral bias, due to its retrospective character. Retrospective randomized controlled trial is suggested to be designed and implemented to analyze the effectiveness of root mesiolingual rotation.

## Conclusions

According to the results of our study, root mesiolingual rotation is an effective method in extractions of horizontal mandibular 3^rd^ molars, especially the deep impacted ones. The 3D surgical simulation based on CBCT reconstruction is valid in surgical design, while precise root bone resistance release using piezosurgery is the key procedure during extraction.
